# Surgery and Physiological Discoveries in Paris—New Method of Treating Fractures—M. Bernard’s Researches—Pancreatic Juice—Bile—Curious Physiological Experiments

**Published:** 1848-08

**Authors:** 


*Surgery and Physiological discoveries in Paris—New method of treating Fractures—M. Bernard's researches—Pancreatic Juice—Bile—Curious Physiological Experiments.*

[The following letter from Prof. March, we copy from the Boston Med. and Surg. Jour., and is addressed to the Editor of that Journal.—Ed.]

My Dear Sir,—Believing that you, and the readers of your Journal, might feel some interest in the new things of the medical profession of Paris, I propose to give you a brief account, or some sketches, of what fell under my observation during a short residence there of five weeks. I will also send you an extract from a letter of deep interest, which I received a few days since from a talented American medical friend, who has been residing in Paris for the last two years.

My time, during the hospital hours of each day, was mostly spent in the surgical wards of the Hotel Dieu, Hopital la Charité and Hopital St. Louis. In Hotel Dieu I saw much of the practice and surgical operations of M. Roux, M. Blandin and M. Boyer (the son of the author of "Boyer's Surgery"). In Hopital la Charité I followed Velpeau pretty generally; though I saw him operate less frequently than the surgeons of Hotel Dieu. In Hopital St. Louis, Jobert is the prominent and leading man in surgery—although Malgaigne, who is a man of genius and of great energy of character, is fast gaining notoriety for his novel mode of treating oblique fractures of the tibia. I will not attempt to give you any account of the numerous operations which I witnessed in the Parisian Hospitals—nor of the good and bad surgical practice which came daily under my observation, as such as has been observed and described again and again by many experienced American surgeons—but will speak only of such points as, to me at least, were new.

Chloroform is universally and very successfully used by the surgeons of Paris; and withal, with more prudence and caution than we Americans are wont to regard the estimate in which human life is held by the bold French surgeons.

M. Jobert, who is perhaps 48 or 50 years of age, and who possesses a very commanding personal appearance, I consider one of the most growing surgeons of Paris. He is perfectly fearless, as his look indicates, and energetic in all his movements. He has a peculiar mode of treating fractures, which consist in adopting the principle of extension and counter-extension *alone*. He uses no *splints* nor *bandages*. He lays the limb on a plain, and somewhat firm surface, with its extremity a little elevated. In fractures of the thigh, or leg, he casts a roller a few times around the ankle and instep, and fixes the foot to the foot-bar of the bed; then a folded sheet or perineal belt is placed under the upper part of the *opposite thigh*, across the perineum and groin, behind the shoulder of the patient, and securely fixed to the head-bar of the bed. The patient is thus confined to his back; but the broken limb receives no pressure, directly or indirectly, in the seat of the fracture, which is perfectly *naked* and exposed for inspection, without any undressing or movement of the limb. I even saw a case of fracture of humerus under the same mode of treatment.

You will probably inquire, with what success was this strange practice attended? I answer, generally pretty favorable. But I now hasten to give something still more new and startling in the practice of M. Malgaigne, to me at least, although it may not be new to you and the readers of your Journal.

One morning, in visiting his ward, I saw a stout, healthy-looking man, of about 40 years of age, lying upon his back, with fracture of the tibia and fibula about five inches above the ankle, which occurred fifteen days before. There was considerable obliquity in the direction of the fracture of the tibia, as was observable by the projecting and overlapping point of the lower extremity of the upper fragment. Inflammation and tumefaction had, in a great degree, subsided; and the limb was resting on a back splint with a foot-piece covered by a cushion, and sustained by lateral cushions and lateral splints—and I believe a cold linseed-meal poultice had been applied to the seat of the fracture, to abate inflammation. All the anterior surface of the limb was naked. So much for the condition of the fracture and the limb, and the apparatus with which it was surrounded, Now for the new and superadded apparatus, and its object:—

The new apparatus consists simply of a steel plate or yoke, moderately semi-lunar, an inch and a half wide, and about eight inches long, with a mortice or fenestrum at each extremity, through which a broad piece of webbing is passed, having on one end a buckle attached, and a *screw pin*, two and a half or three inches long, with a thumb-piece at top, and a sharp bodkin-like point below for half or three fourths of an inch, which passes through a female screw in the centre of the plate or yoke. A handkerchief, or long towel, was passed around the ankle and instep, with the ends extending below the sole of the foot, with which powerful extension was made, while counter-extension was made from the knee, by which the over-lapping point of bone receded, and was readily brought into its proper place. Next, the centre of the yoke, occupied by the *screw pin*, was placed over the upper fragment of the tibia, about an inch and a half from the heretofore projecting part, and the *belt*, made to encircle the base and lateral splints, was tightly buckled; so that the *point* of the *screw pin* was made to perforate the skin on the anterior face of the tibia, at the point above described as being occupied by the yoke. After this, the screw was turned down, so as to make its point enter some little distance into the solid substance of the tibia, which retained the obnoxious fragment in its proper place without extension. The dressing was completed by inserting a few pieces of deal between the lateral splints and the strap, or belt, on the principle of the wedge.

The surgeon stated that he had been greatly troubled in the treatment of oblique fractures of the tiba and fibula, although he had tried all the heretofore known modes of practice; such as *extension* and *counter-extension*—*relaxation of muscles*—*position, elevation, and depression*—and pressure upon the displaced fragment; and that when he first thought of his new mode of treatment, he apprehended that it might be attended with inflammation of the skin, and for aught he knew, *necrosis* or *exostosis* around the pin. "But," he added, "no such thing has happened in a single instance."

I felt a deep interest in watching the result, of at least one case—so that I visited this patient once a week for three weeks. And in confirmation of what the surgeon had previously stated in regard to other cases, not the least inflammation existed even in the skin around the pin; and the limb was in good shape, and appeared to be fast uniting, which he (M. Malgaigne) said would be complete in 30 or 35 days.

The other new thing of which I propose to speak, is physiological; that which relates to the function of the pancreatic juice, in the progress of digestion. I believe there can be no doubt of its being new, since it was not known even to the private pupils of the discoverer, three weeks ago. For the following brief and intelligent description of this new physiological discovery, as well as for some instructive remarks on the result of some surgical cases, the operations for the cure or relief of which, I witnessed before I left Paris, I am indebted to my early friend and pupil, T. W. Powers, M. D., of New York, who is an industrious observer and close thinker; and to whom I am much indebted for many favors received from his hands during my short stay in Paris. I have neither asked nor received his permission thus to use the extract from his letter; nor do I believe it to have been written for the public eye—but the only apology I can offer for the liberty I have taken, is, that it may be the means of doing good.

"I have nothing of much interest to communicate in a professional way. The two cases of vesico-vaginal fistula, which you saw at Jobert's, are doing very well. One is perfectly successful. The case in which you saw Velpeau tie the carotid artery, died from suffocation about thirty hours after the operation. The autopsy showed a great and general dilation of the aorta, aneurism of the innominata, and enlargement of the right subclavin and carotid for an inch or two from their origin. The aneurismal sac projected from the posterior and inner part of the innominata, pressing directly upon the trachea. The sac was about as large as a small hen's or pullet's egg. Velpeau tied the spermatic vein, the other day, in a case of varicocele. Phlebits succeeded the next day, which was followed by purulent absorption, and death. About the same time he amputated the thigh of a man with white swelling of the knee, which terminated fatally; and he ascribed the result to the same cause, viz: purulent absorption.

"The physiologist about whom you inquire, is M. Bernard. He promises to become one of the first experimental physiologists of Europe. He has already highly distinguished himself by his experiments and researches in digestion, and in the circulating and nervous systems. His researches with respect to the pancreatic fluid, are quite recent, and establish, beyond all question, the exact use of that secretion. The followtng is the substance of what he has arrived at on this point. The pancreatic juice, when collected from a living animal (a dog for example,) by means of a fistula artificially established, has nearly identically the same physical character as the saliva, being limpid, colorless, slightly ropy, and rather heavier than water. It is constantly alkaline, and is coagulable by heat and strong acids, owing to the presence of albumen. The saliva is slightly alkaline when collected pure, but never coagulable by heat or acids. When the pancreatic juice is put in contact with azotized aliments, as fibrine, albumen, and gelatine, there is no effect produced. Putrefaction occurs in time, but no digestion. When applied to farinaceous substances, they are changed into sugar which is absorbable. Thus far there is nothing new—all this having been previously established. He, however, has shown—and the *merit* of this discovery is solely due to him—that when this fluid is put in contact with fatty substances of every nature, as oils, animal fats, butter &c., they are quickly digested or decomposed, and reduced to a state in which they may be absorbed into the circulation. This property is peculiar to the pancreatic juice, not being possessed by the saliva, gastric juice, bile, serum, nor any other fluid of the animal economy.

"The pancreas, therefore, now takes rank with the most important organs of the system. I have seen him repeat his experiments with this fluid, and they are quite conclusive. The first effect produced, when you put the pancreatic fluid in contact with oil, or any fatty substance, is to form an intimate emulsion, which will not separate on standing. If you agitate oil with saliva, gastric juice, serum, or *pure* bile, or any other animal fluid, the mixture separates when in repose. (Bile of animals mixes, or makes an emulsion, with grease, by virtue of the pancreatic fluid that is frequently mixed with it.) After the emulsion produced, the oil is decomposed into *glycerine* and a *fatty acid*, as the oleic acid, &c., which are absorbable, as well as the simple emulsion.

"He has also established another very important fact in regard to the digestive fluids—which is, that the union of the bile and the pancreatic fluid produces a new and distinct fluid, having, in addition to the peculiar properties of these two fluids, another superadded, viz: that of digesting azotized substances, or, in other words, the properties of the gastric juice. It therefore digests all alimentary substance, and is altogether the most important of the digestive fluids. This is found in the duodenum in man, below the orifice of the ductus communis choledochus, and in animals below the orifice of the pancreatic duct. By means of this fluid, which he calls the intestinal fluid, aliments which are not digested in the stomach, are acted upon in the intestines. The property that the pancreatic juice possesses of transforming starch into sugar, and which until now has been considered its chief property, is a very subordinate one, and by no means peculiar, as almost all the other fluids of the economy possess it, viz: the saliva, serum of the blood, liquid of cysts, &c.

"All the effects produced by the pancreatic juice, as above described, are equally well seen by taking the pancreas of a freshly killed animal; as a chicken, dog, pig, &c., and bruising it, and pouring a little tepid water upon it. Let it stand, or agitate it a few minutes, and you have an artificial pancreatic fluid, with which you can perform all necessary experiments. If you kill the animal in a state of digestion, the fluid will be more active, as the pancreas is then in a state of greater activity. In the same way you can make artificial gastric juice, by taking the stomach of an animal. But the pancreas must be quite fresh, and the pancreatic fluid changes very quickly and loses its properties, whereas the gastric juice keeps indefinitely."

I am spending a few weeks in "the great metropolis," in sight seeing, with my family, and in visiting its numerous hospitals, and rich pathological museums. I am making, also, further additions to a valuable collection of pathological specimens, which I have procured in Paris for the Albany Medical College. I expect to visit Belgium, Holland, Germany and Switzerland, and if time permits, Scotland and Ireland. If my life be spared, I shall return to America in season to resume my duties in the College, at the commencement of the forthcoming lecture term.

I am very respectfully yours,

LONDON, May 22, 1848. ALDEN MARCH.

